# Hybrid breeding for fall armyworm resistance: Combining ability and hybrid prediction

**DOI:** 10.1111/pbr.13129

**Published:** 2023-07-11

**Authors:** Isaac Kamweru, Yoseph Beyene, Bruce Anani, Victor O. Adetimirin, Boddupalli M. Prasanna, Manje Gowda

**Affiliations:** 1 International Maize and Wheat Improvement Center, Nairobi, Kenya; 2 Life and Earth Sciences Institute (Including Health and Agriculture), Pan African University, Ibadan, Nigeria; 3 Department of Crop and Horticultural Sciences, University of Ibadan, Ibadan, Nigeria

**Keywords:** fall armyworm, general combining ability, hybrid performance, maize, marker-based prediction, native resistance

## Abstract

Fall armyworm (FAW, *Spodoptera frugiperda*) emerged as a major lepidopteran pest destroying maize in sub-Saharan Africa. A diallel mating design was used to generate 210 experimental hybrids from 21 lines. Experimental hybrids and four checks were evaluated in two locations. Commercial checks suffered higher foliar and ear damage compared to the top 15 hybrids. Mean squares associated with the genotypic variation were higher than genotype-by-environment interactions for foliar and ear damage traits. Heritabilities were moderate to high. Significant correlations were observed between grain yield (GY) with ear rot (—0.54) and ear damage (—0.45). Positive and significant GCA effects were observed for GY in seven parental lines, which were developed from multiple insect resistance breeding programmes. CKSBL10153 has the highest GCA value for GY and shows significant GCA effects for foliar and ear damage traits. These lines were identified as the ideal combiners for GY and FAW resistance and are therefore recommended for utilization as testers in the development of FAW-resistant three-way cross-hybrid maize with correlated response for increased GY. GCA and marker-based prediction correlations of GY were 0.79 and 0.96, respectively. Both GCA effects and marker-based models were effective in predicting hybrid performance for FAW resistance.

## 1 | INTRODUCTION

Maize (*Zea mays* L.) is the most productive cereal crop cultivated globally in both tropical and temperate regions for human consumption and livestock feed formulation and as a raw material for many processed goods (Erenstein et al., 2022). For millions of people in sub-Saharan Africa (SSA) who subsist on maize-based diets, maize supplies an estimated energy of 365 Kcal/100 g of their daily cereal consumption (Nuss & Tanumihardjo, 2010). Increased maize production in SSA is expected to bridge the widening gap between the exponentially growing consumer demand and supply (Van Ittersum et al., 2016). Maize productivity is however severely limited by a combination of biotic and abiotic stresses that includes on-farm production constraints, low soil nitrogen, drought, diseases and pests (Baffour Badu-Apraku & Fakorede, 2017). The fall armyworm [FAW; *Spodoptera frugiperda* (J. E. Smith)] insect pest, originally native to the Americas but first reported in Africa by Goergen et al. (2016), has emerged as a major lepidopteran pest affecting maize production in almost all countries in SSA (Feldmann et al., 2019). Depending on the locality and the severity of the infestation, yield losses range from 40% in Honduras (Wyckhuys & O’Neil, 2006), 17% to 72% in Argentina (Murúa et al., 2006) and 21% to 53% in 12 African countries (Day et al., 2017; Overton et al., 2021). The incessant trend of FAW invasion across continents such as Africa, Asia (Lee et al., 2020) and Australia (Maino et al., 2021) precipitates a global food crisis that is projected to diminish gains achieved towards realizing the shared global responsibility of reducing hunger and poverty enshrined in the sustainable development goals blueprint (Fanzo et al., 2021). Coordinated control efforts are therefore required to sustainably mitigate the alarming adverse implications of FAW spread in SSA (Malo & Hore, 2020).

In SSA, the application of synthetic pesticides has been extensively adopted as an emergency response to control FAW devastation (van den Berg et al., 2021). While judicious use of synthetic pesticides to manage FAW damage in the immediate term could be effective, indiscriminate overuse of pesticides to manage high FAW populations leads to the evolution of resistance (Zhang et al., 2020). Furthermore, human and environmental safety concerns have been raised due to the unceasing bioaccumulation of pesticides in the food web, which is harmful to beneficial arthropods (Desneux et al., 2007) and non-targets such as aquatic life (Guo et al., 2008). Transgenic *Bacillus thuringensis* maize with insecticidal activity against FAW is widely cultivated because of its narrow spectrum of activity and high control efficacy (Romeis et al., 2019), but its sustainability has been disputed with the emergence of resistant FAW biotypes (Banerjee et al., 2017). *B. thuringiensis* maize provide significant crop protection against FAW foliar feeding damage (Kavhiza et al., 2022), yet most countries in SSA have prohibitive regulations on handling and cultivation of genetically engineered crops (Nang’ayo et al., 2014). Deployment of host plant resistance (HPR) in managing FAW damage is highly recommended (Kasoma et al., 2022; Matova et al., 2022; Prasanna et al., 2022) since it promotes the sustainability of the agro-ecosystem (Kumar et al., 2022). HPR is also compatible with other integrated pest management approaches (Dara, 2019) such as the use of pesticides (Phambala et al., 2020), transgenics (Botha et al., 2019), natural predators (Koffi et al., 2020; Tendeng et al., 2019) and cultural control practices (Harrison et al., 2019).

In the Americas, morphological traits have been used to identify a series of FAW-resistant genotypes (Widstrom et al., 1992), and the resistant lines were utilized as donors to improve elite but susceptible lines (Williams et al., 1978). Even though most resistance breeding pipelines in SSA rely on conventional breeding, scientific evidence on tropical maize germplasm endowed with intrinsic resistance to FAW foliar feeding is narrowly restricted in scope and outlook (Prasanna et al., 2018). This is partly because FAW is an invasive pest, and the focus of insect resistance breeding was on existing lepidopteran pests such as stem borers. Development, deployment and improved FAW-resistant hybrids are the pre-requisites for a better farmer harvest, trade and financial surplus in the context of a devastating pest that is likely to establish itself as a multi-generation pest of economic importance in SSA (Senay et al., 2022). In addition to screening large sets of diverse maize germplasm under artificial FAW infestation to discover resistance lines from multiple sources (Soujanya et al., 2022), determination of their combining abilities of these inbreds with a view of quantifying their genetic effects controlling the inheritance of the resistance is also crucial (Fasahat et al., 2016). Further multi-location evaluation of all hybrids facilitates selection of the promising hybrids.

With the current advances in doubled haploid (DH) technology, a high number of homozygous elite inbred lines are available for use in developing superior hybrids. In programs where breeding resources are inadequate, conducting multi-environment trials has practical limitations (Wang, Wang, et al., 2020). The ability to predict hybrid performance based on the general combining ability (GCA) or marker data of the parental lines could be used to circumvent the limitations of phenotypic evaluation in multiple environments. A comparison of GCA and marker-based prediction models indicates that hybrid performance can be predicted with high accuracy (Kamweru et al., 2023). Several studies noted that prediction of hybrid performance promotes the optimal use of breeding resources, improves the efficiency of inbred line selection in hybrid maize breeding pipelines, and holds potential value in accelerating the genetic gains in crop improvement (Kadam et al., 2016; Technow et al., 2014; Vélez-Torres et al., 2018). Predicting the performance of untested hybrids or missing combinations in FAW resistance breeding could therefore accelerate the development of high-yielding, FAW-resistant varieties for commercial release. The objectives of the present study were to (i) estimate the effects of the GCA and SCA for resistance to FAW in tropical maize lines with an aim to identify the parental lines that are good combiners for both FAW resistance and superior yield and (ii) investigate the reliability of GCA and genomic enabled model in predicting hybrid performance.

## 2 | MATERIALS AND METHODS

### 2.1 | Plant material

For this study, 21 parental lines (Table 1) were selected from diverse populations maintained by CIMMYT. These lines were known to be tolerant to FAW, stem borers, multiple insect pests, resistance to maize lethal necrosis (MLN) and foliar diseases, better per se performance under optimum and drought conditions.

### 2.2 | Experimental design and trial sites

A partial diallel design was used to generate 210 experimental hybrids (Table S1) from 21 parental lines. All hybrids were evaluated for two seasons together with four widely grown commercial checks (DH04, DK8031, Duma43, WE1101) under artificial FAW infestation in Kiboko (20°15′S, 37°75′E, 975 masl) and natural FAW infestation in Kakamega (0°16′S, 34°42′E, 1585 masl) where severe FAW infestation was observed. The experiments were laid in an α-lattice design with two replications per site. The row-to-row distance was 0.75 m in both sites. Two seeds were planted at intervals of 0.25 m. Plants were thinned to one plant per hill 3 weeks after emergence to obtain a final population density of 53,333 plants per hectare. Standard agronomic management practices were followed for proper crop growth and development.

### 2.3 | FAW infestation and data collection

The artificial diet described by Tefera et al. (2011) was used to rear FAW neonates under ambient laboratory conditions (temperature of 27 ± 1°C, 12:12 h light/dark photoperiod and relative humidity of 75 ± 5%) at the Kenya Agricultural and Livestock Research Organization (KALRO) insectary located at Katumani (1°35′S, 37°15′E, 1610 masl) in Kenya. FAW infestation was implemented manually by applying eight first instar FAW larvae to the furl and whorl leaves of each plant using a painter brush. All plots were artificially infested on the same day during the V3 phenological stage of maize growth and development (Nielsen, 2014). Foliar damage (FD) was visually rated for each plant per plot at 7, 14 and 21 days after artificial FAW infestation. In Kakamega, natural infestation of FAW was severe; therefore, like artificial infestation conditions in Kiboko, in Kakamega, also three FD scores were recorded at three times with 7 days of interval. A visual rating scale of 1–9 as described earlier in FAW integrated pest management guide by Prasanna et al. (2018) was used to score leaf feeding damage. On this scale, 1 = *no visible damage*, plants are considered as resistant, and 9 = *severe damage*, most leaves with long lesions and complete defoliation (Kamweru et al., 2023). In addition, other agronomic traits were also recorded like days to anthesis (AD, days from planting to 50% pollen shed), silking days (SD, days from planting to 50% silking), anthesis to silking interval (ASI), plant height (PH), distance in cm from the ground to the top of the tassel, ear height (EH, distance in cm from the base of the plant to the main ear bearing node). Ear damage (ED) was rated on a scale of 1–9, where briefly 1 = *no visible damage to the ear* and 9 = *90%*–*100% damage to an ear* (Kamweru et al., 2023). GY was obtained from the shelled grain weight per plot, converted to tons per hectare (t/ha) after adjusting moisture content to 12.5%. After defoliating the maize stalks, the number of FAW exit holes (EXHL) was counted for each plant per plot. Tunnelling length (TLGTH) per plant per plot was measured in millimetres. The traits EXHL and TLGTH data were collected only in an artificially inoculated location.

### 2.4 | Statistical analysis

FAW FD and ED scores were based on an ordinal scale; therefore, data were checked for statistical model fitting, that is, normally distributed, constant variance and independent (Rawlings et al., 1998). A plot of residuals against fitted values has shown that the residuals were symmetrically distributed with constant variance; thus, the data were not transformed. Detected outliers were removed from the analysis. Best linear unbiased predictions (BLUPs) and best linear unbiased estimates (BLUEs) for each hybrid were generated using Multi-Environment Trait Analysis software in R environment (Alvarado et al., 2015). Analysis of variance (ANOVA) was conducted using a general linear model (GLM) in R environment (R Development Core Team, 2019) on trait data collected from each site and pooled data from across environments. In this model, genotypes were considered as fixed factors, while replications, blocks, and test environments were taken as random factors. Parental line GCA and SCA effects of F_1_ hybrids as well as their mean squares under each and across environment were computed using Griffing’s linear model 1 and method 4 for analysing diallel crosses (Griffing, 1956). Commercial checks used were excluded from the diallel analysis. The following model was used to analyse data in AGD-R (Analysis of genetic designs in R environment) software, version 4.0 (Rodríguez et al., 2015).

Yii'k=μ+Ek+Gi+Gi'+Sii'+(EG)ik+(EG)jk+(ES)ii'k+eii'k

Where ${\text{Y}}_{ii}{}_{´k}$ is the performance of the single cross hybrid $\left(i_{\text{x}}i´\right)$ in the *k*th environment; μ is the overall mean; ${\text{E}}_{k}$ is the *k*th environment effect; ${\text{G}}_{i}$, ${\text{G}}_{i'}$, and ${\text{S}}_{ii}{}_{´}$ are GCA and SCA effects (Griffing, 1956); ${\left(\text{EG}\right)}_{ik}$ and ${\left(\text{EG}\right)}_{i'k}$ are GCA effects of i and i' parents and their interaction with the environment, respectively; (ES)ii'k is hybrid interaction with the environment; and eii'k is the error term. Mean squares for hybrid and environment were tested against the mean squares for genotype-by-environment (G×E) interaction as error terms, while G×E mean squares were tested against the pooled error. Similarly, the significance of GCA and SCA sources of variation was determined using the corresponding interactions with the environments as error terms. Mean squares for combining abilities were computed based on entry means, and subsequently, the error mean squares used to test the significance of GCA and SCA interactions with the environment were obtained by dividing the pooled error mean squares from the ANOVA by the number of replications (Dabholkar, 1999; Ferreira et al., 2004; Griffing, 1956). Significance of GCA and SCA effects was tested using the *t*-statistic. For each trait, the baker ratio (Baker, 1978) was used to evaluate the relative importance of GCA and SCA. The ratio of phenotypic variance contributed by the genetic variance was used to compute broad-sense heritability based on the entry means (Hallauer et al., 2010). The predictability of hybrid performance was considered high if the ratio of GCA effects (2GCA) to total genetic effects (2GCA + SCA) was closer to unity (Hung & Holland, 2012). Pearson’s correlation coefficients were calculated between pairs of agronomic traits using BLUPs from across environments. A leave-one-hybrid-out cross-validation procedure described by Schrag et al. (2009) was implemented to determine the coefficients of the correlations between F_1_ hybrids and the sum of GCA effects of both parents r (GCA, F_1_P).

All parental lines were genotyped using the Diversity Arrays Technology marker platform (Jaccoud et al., 2001), and 36,667 SNPs evenly distributed across the 10 maize chromosomes were used. Trait Analysis by aSSociation, Evolution, and Linkage (TASSEL) software ver. 5.0 (Bradbury et al., 2007) was used to implement stringent quality control criteria and 11,253 high-quality SNPs with minor allele frequency >0.05 and <10% missing values were retained for use in marker-based prediction of hybrid performance.

Prediction of hybrid performance was implemented in this study and described earlier by Technow et al. (2014). The marker-based prediction model was fitted using the Bayesian Generalized Linear Regression (BGLR) package in R environment (Pérez & De Los Campos, 2014) and utilized fivefold cross-validations to quantify prediction accuracy.

## 3 | RESULTS

### 3.1 | Performance of single-cross hybrids

In 2019, the presence of FAW insect pest population was so high in natural condition that the FD was comparable with FD under artificial infestation. FD scores and ED scores from each location indicated comparable pest pressures across test locations (Figure S1), which resulted in significant (*P<* 0.01) Pearson correlation coefficients among phenotypic values determined between the environments (Table S2). This suggested combined analysis across environments should not be severely biased.

**FIGURE 1:**
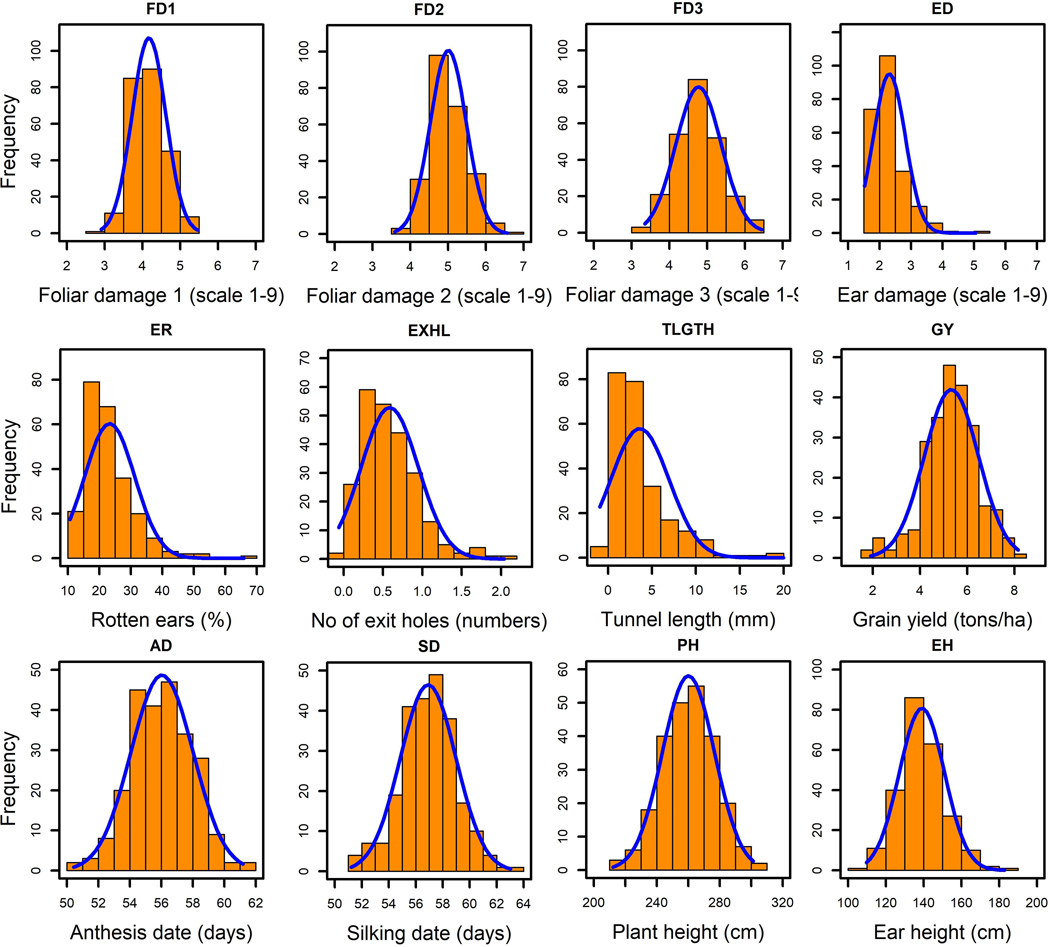
Frequency distribution of the means for foliar damage scores 7, 14, and 21 days after FAW infestation (FD1, FD2 and FD3, respectively), grain yield (GY), ear damage (ED), rotten ears (ER), days to anthesis (AD), days to silking (SD), plant height (PH) and ear height (EH).

The phenotypic data for FAW resistance traits and other agronomic traits showed normal distribution (Figure 1) except for ED where data were skewed towards resistance. Leaf injury ratings (FD1, FD2, FD3) were continuously distributed from the resistance to the susceptible range. Results showed that the germplasm under study exhibited quantitative resistance to FAW foliar feeding damage. FD3 values revealed 25 hybrids with score of <4 in 1–9 scale indicating the availability of diverse sources of resistance to FD. Three hybrids CKSBL10008xCM338, CKSBL10039xCKIR04005 and CKSBL10008xCML71 had the least FD scores and were identified as good sources of leaf feeding resistance.

The mean performance of all hybrids across environments was 5.32 t/ha (range = 1.90–8.16 t/ha), whereas the mean performance of best 15 hybrids was 7.45 t/ha, which is far higher than best commercial check (WE1101, 5.29 t/ha; Table 2). The experimental hybrids CKSBL10153xCKDKL0214, CKSBL10039xCKSBL10060 and CKSBL10011xCLRCY039 with respective GY means of 8.16, 7.87 and 7.60 t/ha were the highest-yielding hybrids (Table 2). WE1101 was the best commercial check that outperformed 44% of the experimental hybrids. Among all the experimental hybrids, 95, 4 and 25 hybrids exhibited FD scores of <4 for FD1, FD2 and FD3, respectively. The mean performance of the best 15 hybrids was 4.12, 4.90 and 4.51 for FD1, FD2 and FD3, respectively, whereas for the commercial checks, mean performance was 4.78, 5.85 and 5.67 for FD1, FD2 and FD3, respectively, which is higher than mean performance of experimental hybrids. FD ratings across the three scoring assessments indicate that commercial checks on average suffered 14% more compared to the best 15 hybrids. FAW-inflicted ED was 80% more among all checks compared to the mean of the top 15 hybrids. Fewer number of exit holes (0.6 vs. 2), shorter cumulative tunnelling length (3 vs. 14 mm) and a lower percentage of rotten ears (3.7 vs. 34%) were also observed on average among the top 15 high-yielding hybrids when compared to the average of commercial checks.

### 3.2 | Trait correlations

**FIGURE 2:**
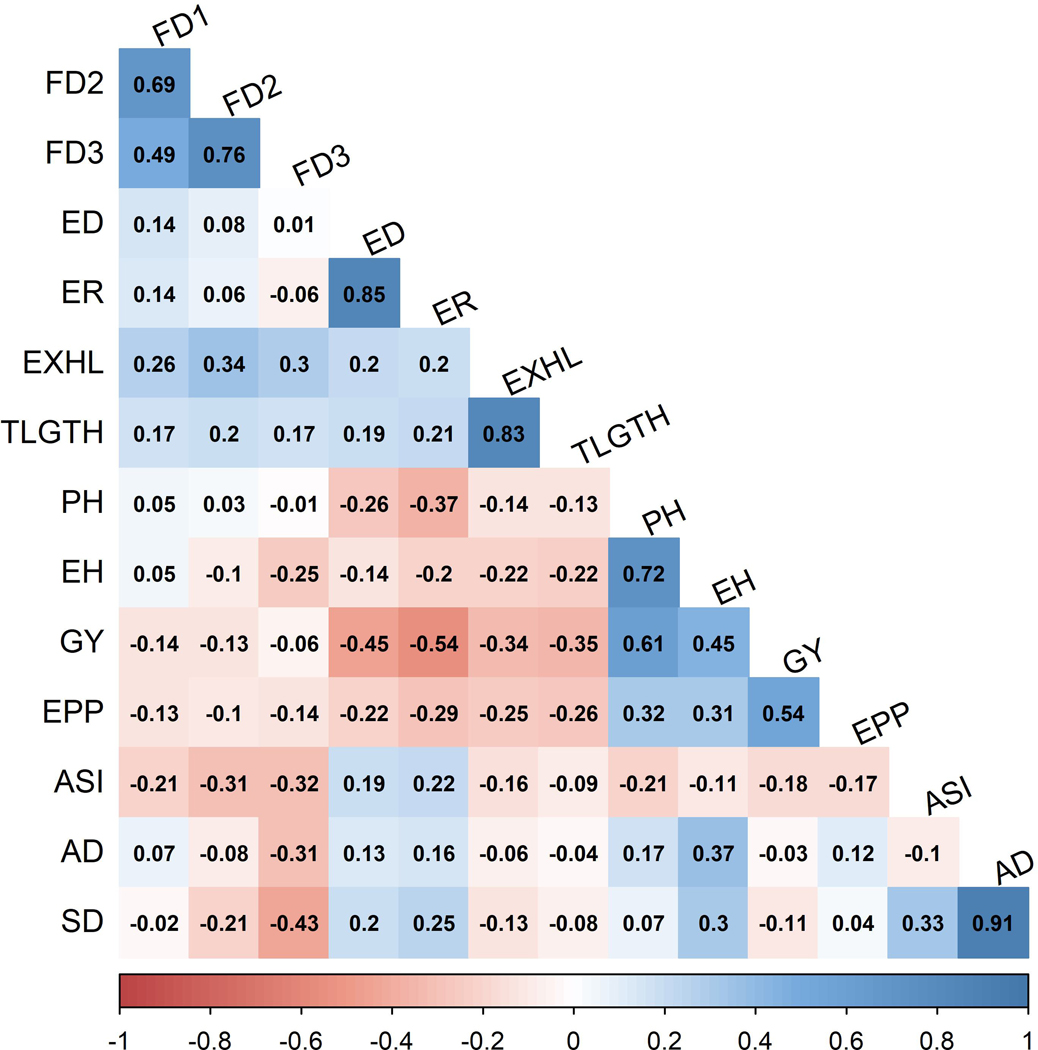
Phenotypic correlations between traits evaluated under FAW-infested conditions. The correlation level is colour-coded according to the colour key indicated on the scale. Correlations with >0.15 and >0.19 were significant at 0.05 and 0.01 levels, respectively. AD, days to anthesis; ASI, anthesis to silking interval; ED, ear damage; EH, ear height; EPP, ears per plant; ER, number of rotten ears in (%); EXHL, number of exit holes; FD1, FD2, and FD3, mean leaf damage scores at different time interval; GY, grain yield (tha^−1^); PH, plant height; SD, days to silking; TLGTH, cumulative tunnelling length.

All traits except FD1, FD2, FD3, ASI and AD were significantly correlated with GY (Figure 2). Positive and significant correlations were observed between GY and EPP (r = 0.54), GY and PH (r = 0.61) and GY and EH (r = 0.45). High magnitudes of negative but significant correlations were observed between GY and both the percentage of rotten ears (r= −0.54) and ED (r = −0.45). Traits such as AD and SD (r = 0.91), PH and EH (r = 0.72), percentage of rotten ears and ED (r = 0.85), EXHL and the cumulative tunnelling length (r = 0.83) exhibited strong positive correlations. FD3 was negatively and significantly correlated with SD, AD, ASI and EH. Highly positive and significant correlations were observed between FD1, FD2, FD3 and EXHL (Figure 2).

### 3.3 | Analysis of variance

Across environment analysis showed significant mean squares for GY and all other FAW resistance traits for both genotype and G×E interactions (Table 3). The mean squares of GCA and SCA and their interactions were also significant for GY, FAW resistance traits and other agronomic traits. The broad-sense heritabilities were high for AD, SD, EH and PH (range = 0.72–0.84), moderate for GY, FD3 and ED (range = 0.41–0.53) and low for FD1 and FD2 (0.25–0.33). The estimates of variance components for GY, FAW resistance traits and agronomic traits obtained during combining ability analyses are summarized in Table 4. The GCA variances $\left(\sigma^{2}{}_{\text{GCA}}\right)$ were higher than SCA variances $\left(\sigma^{2}{}_{\text{SCA}}\right)$ for FD1, FD3, ED, AD, SD, PH and EH, whereas SCA variances were higher than GCA variances for FD1 and GY. Additive variances $\left(\sigma^{2}{}_{\text{A}}\right)$ were higher than dominance variance $\left(\sigma^{2}{}_{\text{D}}\right)$ for all the traits. The baker ratio shows values showed >0.70 for all the traits except FD2 and GY where SCA is equally predominant as of GCA.

### 3.4 | GCA effects

The GCA effects of the inbred lines were used to detect good combiners for various traits such as GY and resistance to FAW FD and ED traits. Our results revealed that 10 among the 21 parents had positive GCA values for GY with higher values associated with line CKSBL10153 (Table 5). For FAW resistance traits, negative GCA values are preferred since lower score represents resistance to FAW. The negative and significant GCA values were observed for 11, 10 and 11 parents for FD1, FD2 and FD3, respectively. Among the 21 parental lines, CML71, CKSBL10153, CKIR04005, CKSBL10008, CKSBL10039, CKSBL10011 and CKSBL10026 showed significant and negative favourable GCA effects for FD1, FD2, FD3 and ED. In addition to showing positive GCA values for GY, parental lines CKSBL10153, CLRCY039, CKSBL10039 and CKSBL10008 had desirable GCA values for resistance to FD (FD1, FD2 and FD3) and ED.

### 3.5 | Prediction of hybrid performance

**FIGURE 3:**
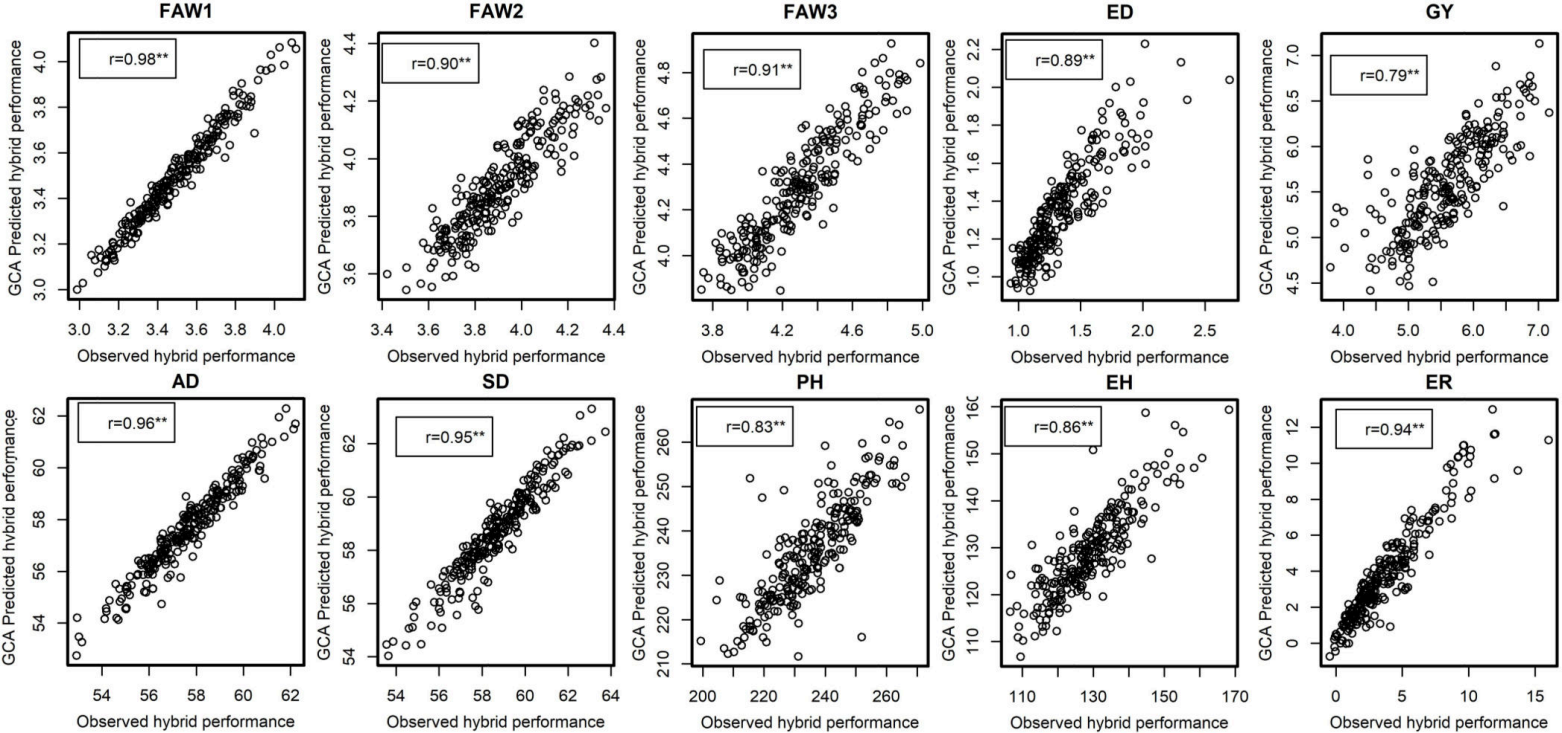
Leave-one-hybrid-out cross-validated r values between general combining ability (GCA)-based predicted hybrid performance and observed hybrid performance for grain yield and other FAW and agronomic traits **Significant at the 0.01 probability level. AD, days to anthesis; FD1, FD2, FD3, mean foliar damage scores at three different time intervals; GY, grain yield; PH, plant height.

**FIGURE 4:**
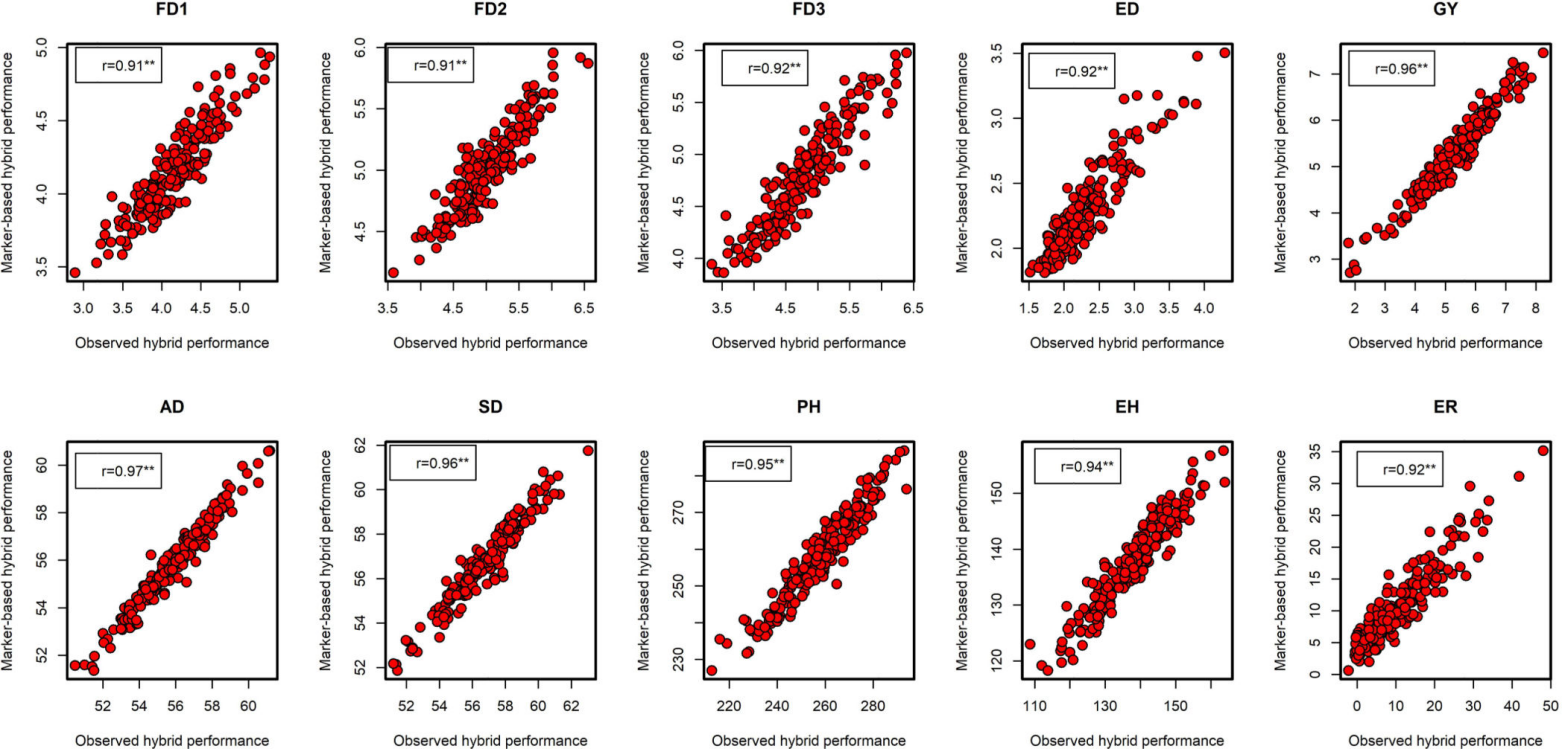
The correlation between marker-based predicted and observed F1 hybrid performance for grain yield and other agronomic traits evaluated under FAW infestation in two environments. **Significant at the 0.01 probability level. ED, ear damage; EH, ear height; ER, ear rot; FD1, FD2, FD3, mean leaf damage scores at three different time intervals; GY, grain yield; PH, plant height; SD, silking date.

The correlation between GCA predicted and observed field performance of the hybrids was high for traits such as FD1 (r = 0.98, *P* < 0.01), FD2 (r = 0.90, *P* < 0.01), FD3 (r = 0.91, *P* < 0.01), AD (r = 0.96, *P* < 0.01), SD (r = 0.95, *P* < 0.01), ED (r = 0.92, *P* < 0.01), ER (r = 0.93, *P* < 0.01), EH (r = 0.86, *P* < 0.01) and PH (r = 0.83, *P* < 0.01). GY had the lowest correlation coefficient (r = 0.79, *P* < 0.01) (Figure 3). The correlation between marker predicted and observed field performance of the hybrids was also high for all traits: FD1 (r = 0.91, *P* < 0.01), FD2 (r = 0.91, *P* < 0.01), FD3 (r = 0.92, *P* < 0.01), AD (r = 0.97, *P* < 0.01), SD (r = 0.96, *P* < 0.01) ED (r = 0.92, *P* < 0.01), ER (r = 0.92, *P* < 0.01), EH (r = 0.94, *P* < 0.01), PH (r = 0.95, *P* < 0.01) and GY (r = 0.96, *P* < 0.01) (Figure 4). Both GCA and marker-based predictions were reliable in predicting hybrid performance.

## 4 | DISCUSSION

In the absence of a sustainable FAW control strategy, maize production in SSA is projected to be exceptionally risky (Haftay, 2020; Overton et al., 2021). This is because climatic conditions in most maize-growing agro-ecology support the multi-generational establishment of voracious and polyphagous FAW pest (Senay et al., 2022). Effective deployment of HPR to mitigate yield and economic losses emanating from the wide-scale destruction of the maize crop by the FAW insect pest, therefore, requires an in-depth understanding of the genetic differences for resistance to FD and other important traits like GY. Our results showed that under FAW-infested conditions, many of experimental hybrids evaluated in this study expressed resistance to tolerance reaction to FAW foliar feeding damage (Table 2). Breeding for FAW resistance is challenging due to the paucity of highly resistant germplasm and known FAW’s propensity of developing resistance to various control tactics (Storer et al., 2012). The best yielding hybrids under FAW-infested conditions includes CKSBL10153xCKDHL0214 and CKSBL10039xCKSBL10060, and other 13 hybrids listed in Table 2 have high utility in tropical maize breeding programmes that emphasize insect resistance breeding along with increased yield potential. Interestingly, the parental lines of these hybrids were also known to be resistance for maize stem borers (Table 1). In tropical hybrid maize breeding in SSA, the most popular final products are the three-way cross hybrids due to their lower seed production cost (Tripathi et al., 2020). This study, therefore, recommends the utilization of the best 10 single cross hybrids identified in this study as testers in the development of three-way cross hybrids. Due to recombination, the frequency of favourable alleles conferring FAW resistance in three-way cross hybrids is expected to be high compared to that of single-cross hybrids (Park et al., 2011). Combining the FAW resistance trait from multiple germplasm sources could help improve the durability and stability of the resistance trait.

Significant mean squares associated with the genotype source of variation were observed for all the traits (Table 3). This suggested there was an adequate amount of genetic diversity in the set of germplasm under study. High heritability estimates ranging from 0.72 to 0.84 observed for AD, SD, EH and PH traits suggested the presence of large genetic variability among hybrids and indicated that selection based on these traits could be effective. It could also be argued that a few major genes controlled these traits. Low heritability estimates observed for traits such as FD1, FD2 and ER (range 0.25–0.33) could be attributed to high magnitude of hybrid-by-environment interaction effects (De Souza et al., 1998). In the current study, low heritability estimates corresponded with significant G × E interaction variance. It is important to note however that heritability estimates reported in this study are population specific and measure the proportion of phenotypic variance that is a result of genetic factors.

The desirability of the relationships between traits was examined to identify traits that are positively correlated to GY. Positive and significant correlations were observed between GY and EPP (r = 0.54), GY and PH (r = 0.32) and GY and EH (r = 0.31). This suggested that EPP could be used as an indirect selection index to increase GY. Correlations between GY and FD scores were negative. Similarly, yield losses due to FAW infestation have been attributed to extensive defoliation that inhibits photosynthetic output as well as mechanical wounding that alters the normal functioning of the remaining leaf tissue (Anjorin et al., 2022). Leaf injury in tomato plants infested with the sweet potato whitefly was associated with reduced gaseous exchange (Buntin et al., 1993), and low leaf photosynthetic productivity has been reported in two host plant species colonized by the sapsucking first instar nymphs of *Coccus hesperidium* L. (Golan et al., 2015). A significantly negative correlation was observed between GY and tunnelling length (r = −0.35**). This suggested that nutrient uptake by the host plant was disrupted by FAW tunnelling and this also contributed to reduction in GY. Host plant nutritional deficiency due to insect pest infestation was similarly reported by Heng-Moss et al. (2003). During the reproductive maize stages, FAW larva burrows the husk as an adaptation to increase its chances of feeding on the fruit of the maturing crop and safeguard itself from any harm caused by natural predators, insecticides and lethal concentrations of insecticidal proteins expressed by leaf tissues (Pannuti et al., 2016; Paula-Moraes et al., 2012; Perkins et al., 2008).

In the current study, a high magnitude of negative but significant correlations was observed between GY and both ear rot (r = −0.54) and ED (r = −0.45). According to Womack et al. (2018), the presence of larval frass on FAW-damaged kernels creates conditions that favour the growth of mould pathogens such as *Fusarium* and *Aspergillus flavus* that predispose maize to fungal attacks, rots and mycotoxin accumulation, which adversely affect the quality and quantity of maize GY. In FAW-infested maize seed multiplication sites, FAW-induced ear rot could also cause the unavailability of seeds to farmers. Limited access to planting materials could result in food price increases along with hunger and rural poverty. A positive correlation observed between FAW-inflicted ED and the percentage of rotten ears (r = 0.85) suggested that selection for reduced ED would simultaneously lead to a reduction in the number of rotten ears. The conclusive overview is that examination of the direction and the magnitude of correlated responses could benefit multiple trait selection conducted in resistance breeding. On the contrary, the correlations between all FD scores and ED are not significant, which supports possibly these two traits are governed independently by different genetic factors, so, improving resistance to these traits also not inter-dependent. Similar non-significant correlations were also observed in an association panel (Kamweru et al., 2022).

For breeders, GCA values provide important decision-making input information to selecting parents or suitable inbred testers for use in hybrid breeding. Positive and significant GCA values for GY were observed for seven parental lines (CLRCY039, CKSBL10153, CKSBL10008, CKSBL10039, CKSBL10060, CKDHL0214 and CKDHL164271); four are developed from multiple insect resistance breeding program specifically lines for stem borers (SBL). CKSBL10153 has the highest GCA value for GY and is also showing significant GCA effects for FD1, FD2, FD3 and ED, which makes this line one of the promising donors to improve FAW resistance as well as GY. In addition, three lines CKSBL10039, CKSBL10008 and CLRCY039 also showed a significant and high frequency of favorable alleles or GCA effects for GY, FD, and ED (Table 5). All seven parental lines were found in various cross combinations among the top-yielding hybrids suggesting their substantial contribution in better GY performance under FAW infestation. Line CKSBL10153, which had the highest GCA effects for GY is contributed in three of the top 15 hybrids, whereas CLRCY039, CKSBL10039 and CKDHL0214 contributed to four combinations (Table 2). Each of parents CKSBL10153 and CKSBL10008 appeared in three hybrid combinations among the top 15 hybrids, while parent CKSBL10060 was found in two cross combinations. Eleven parental lines with significant and negative GCA values for either FD1, FD2, FD3 and ED traits were identified. These lines had a high frequency of favourable alleles that conferred resistance to FAW foliar feeding damage and ED. Similarly, resistance to both ear rot and ED was conferred by favourable alleles found in 10 parental lines. Notably, parents CKSBL10153, CLRCY039, CKSBL10008 and CKL10060 exhibited desirable attributes for both GY and FAW resistance indicator traits such as FD1, FD2, FD3, ear rot and ED. Moose and Mumm (2008) noted that intermating promising lines increase the frequency of the favourable alleles in breeding populations. Correspondingly, hybrid combinations involving these parental lines ought to be developed to optimize FAW resistance among hybrids and produce superior-yielding progenies that are also resistant or tolerant to FAW. These four parental lines were identified as the ideal combiners for GY and FAW resistance and are therefore recommended for utilization as testers in the development of FAW-resistant three-way cross-hybrid maize with a correlated response for increased grain yield. Contrary to the results of previous studies (Butron et al., 2002; Klenke et al., 1986; Nyhus et al., 1989), selection for FAW resistance in this study was not associated with a yield penalty. Parental lines with large positive GCA values for FD1, FD2, FD3, ear rot, ED, EXHL and TLGTH traits suggested either they were susceptible to FAW infestation or reducing the level of tolerance for FAW infestation when they are used as parents in hybrid combination. Among 210 hybrid combinations, 37 hybrids revealed significant SCA effects for GY including 17 of them with significant and positive effects.

In the current study, GCA and marker-based prediction correlations of the GY were 0.79 and 0.96, respectively (Figures 3 and 4). These predictions were higher than those reported by Zhang et al. (2022), Wang, Zhang, et al. (2020) and Kamweru et al. (2023). Although GCA effects were sufficiently reliable in predicting hybrid performance, marker-based predictions were consistently higher for complex traits like GY, which suggested that prediction models that incorporate marker data are more effective. Our findings are consistent with reports of Pérez et al. (2010) and Crossa et al. (2010), indicating that marker-based models were more precise in predicting complex traits like GY. Our results revealed high correlation coefficients between predicted and observed values for moderately complex traits like PH and EH with high heritability estimates. Moderately low prediction accuracy for complex traits with low heritability such as GY reported previously by Zhang et al. (2015) corroborates with our findings. Several studies seem to agree that predicting the performance of untested germplasm using marker-based models could accelerate the realization of genetic gains per unit cost and time (Bernardo, 2021; Beyene et al., 2015; Windhausen et al., 2012). According to Bhat et al. (2016), the feasibility of incorporating predictive breeding in most breeding pipelines is high due to the cost-effectiveness of most genotyping techniques. In contrast, for FAW resistance traits like FD and ED, GCA-based prediction correlations were higher than marker-based correlations, which supports GCA-based predictions alone are good enough to choose appropriate parents for hybrid combinations. However, for GCA-based predictions, prior knowledge on GCA effects of the lines is required, which is laborious, and this is where marker-based prediction is advantageous over GCA-based predictions. Nevertheless, for FD and ED traits, both GCA-and marker-based predictions are effective and can be used in FAW resistance hybrid breeding.

## 5 | CONCLUSION

Detection of significant genotypic variation in the set of maize germplasm evaluated promotes prospects of improving grain yield and FAW resistance traits in tropical maize. Although FD scores increased over time, levels of susceptibility varied from early to mid-whorl growth stages. Experimental hybrids that showed low levels of FD and ED, fewer exit holes and shorter cumulative tunnelling length along with high GY were identified as suitable parents that could be used in the development of three-way cross hybrids to improve maize-based food security in FAW endemic agro-ecologies. Genotypes that showed favourable GCA estimates for both GY and FAW resistance indicator traits were vastly adapted to FAW-infested conditions and could be useful in the deployment of native genetic resistance to FAW. Both additive and non-additive effects were important in regulating the inheritance of GY under FAW infestation, while additive genetic effects were important for the inheritance of FD, ear rot and ED. Considering that both additive and non-additive gene actions were important in conditioning FAW resistance in maize, a recurrent selection that exploits additive variance and a heterosis selection that exploits transgressive segregation could therefore be utilized to improve these traits. GCA-based predictions are promising and can be effectively used to select appropriate lines for hybrid combinations. However prior information on GCA effects of the lines is required, which is resource intensive. To accelerate gains in the selection, the integration of marker-based prediction models into existing resistance breeding pipelines is vital.

## Supplementary Material

Supplementary Figures and Tables

## Figures and Tables

**TABLE 1:** Description of the 21 inbred parents used in making 210 F_1_ hybrids used in the study.

No.	Genotype	Parental status	Grain colour	Attribute
1	CKDHL0214(CML567)	Inbred line	White	Drought tolerance, FAW tolerance
2	CKDHL121320	Inbred line	White	FAW tolerance
3	CKDHL164271	Inbred line	White	FAW tolerance
4	CKDHL166075	Inbred line	White	FAW tolerance
5	CKIR04005	OPV	White	Multiple insect resistance
6	CKSBL10008	Inbred line	White	Stem borer tolerance, tolerance to FAW, GLS and TLB
7	CKSBL10011	Inbred line	White	Stem borer tolerance, FAW tolerance
8	CKSBL10020	Inbred line	White	Stem borer tolerance, FAW tolerance
9	CKSBL10026	Inbred line	White	Stem borer tolerance, FAW tolerance
10	CKSBL10027	Inbred line	White	Stem borer tolerance, FAW tolerance
11	CKSBL10039	Inbred line	White	Stem borer tolerance, FAW tolerance
12	CKSBL10043	Inbred line	White	Stem borer tolerance, FAW tolerance
13	CKSBL10060	Inbred line	White	Stem borer tolerance, FAW tolerance
14	CKSBL10153	Inbred line	White	Stem borer tolerance, FAW tolerance
15	CKSPL10007	Inbred line	White	MLN resistance, FAW tolerance
16	CKSPL10089	Inbred line	White	Insect tolerance, FAW tolerance
17	CKSPL10158	Inbred line	White	Insect tolerance, FAW tolerance
18	CLRCY039 (CML574)	Inbred line	Yellow	Tolerance to FAW, MLN, tar spot complex and drought
19	CML22	Inbred line	White	Tolerance to FAW and ear rot
20	CML71	Inbred line	Yellow	Tolerance to FAW and southwestern corn borer and ear rot
21	CML560	Inbred line	White	Stem borer resistant, FAW tolerance

**TABLE 2:** Mean performance of the top 15 F_1_ experimental hybrids, commercial checks and their mean performance for GY and FAW resistance traits.

Genotype	GY (t/ha)	AD (days)	PH (cm)	FD1 (1–9)	FD2 (1–9)	FD3 (1–9)	ED (1–9)	ER (%)
CKSBL10153/CKDHL0214	8.16	58.71	273.92	3.87	5.46	4.39	1.96	5.33
CKSBL10039/CKSBL10060	7.87	55.60	279.81	4.35	4.93	4.79	1.81	2.04
CKSBL10011/CLRCY039	7.60	57.83	275.11	4.27	4.98	4.76	1.85	−0.43
CKDHL164271/CML338	7.56	54.40	280.50	4.78	5.72	5.10	1.78	3.64
CKDHL0214/CLRCY039	7.52	59.57	263.95	4.29	5.47	5.22	1.99	2.74
CKSBL10039/CKSBL10153	7.51	58.25	289.92	3.87	4.03	3.61	2.00	12.00
CKSBL10043/CKSBL10060	7.47	52.57	245.47	3.69	4.36	4.76	1.79	1.45
CKSBL10008/CML338	7.42	54.30	281.35	2.91	3.59	3.35	2.01	2.04
CML560/CKSBL10008	7.42	54.35	274.88	4.42	5.59	4.93	2.20	3.94
CKDHL0214/CML22	7.41	58.39	263.14	4.63	4.95	4.72	2.38	3.17
CKSBL10008/CLRCY039	7.26	60.58	272.28	4.11	4.53	4.20	1.78	2.17
CKSBL10153/CLRCY039	7.17	61.18	278.15	3.72	4.55	3.81	1.73	−0.10
CKSBL10039/CLRCY039	7.16	59.96	285.15	4.54	5.19	4.59	2.49	5.45
CKDHL0214/CML338	7.14	55.26	275.24	4.36	5.25	4.63	2.01	3.39
CKSBL10039/CML338	7.08	55.60	267.80	3.99	4.83	4.74	1.85	8.12
WE1101	5.29	56.56	258.55	4.68	5.45	5.32	2.57	8.59
Duma43	4.40	53.53	266.79	4.41	5.58	5.55	3.85	35.99
DK8031	3.69	55.68	260.68	4.99	6.03	5.75	4.65	50.64
DH04	2.97	58.01	254.36	5.03	6.32	6.07	3.31	40.83
Mean of the checks	4.08	55.95	260.10	4.78	5.85	5.67	3.59	34.01
Mean of best 15 hybrids	7.45	57.10	273.78	4.12	4.90	4.51	1.97	3.66
Mean of all hybrids	5.32	56.04	260.06	4.17	5.00	4.78	2.31	12.37

Abbreviations: AD, days to anthesis; ED, ear damage; ER, ear rot; FD1, FD2 and FD3, foliar damage scores at three different time intervals; GY, grain yield; PH, plant height.

**TABLE 3:** Mean squares for grain yield, FAW resistance associated traits and other agronomic traits of 210 medium maturing tropical maize hybrids evaluated in diallel experiment under FAW infestations in two locations in Kenya.

Source	DF	FD1	FD2	FD3	ED	GY	AD	SD	PH	EH
Environment, E	1	585.14**	1226.48**	188.82**	904.29**	517.27**	1370.63**	1643.60**	449,354.88**	66,285.68**
Rep (Env), R	2	11.15**	0.70*	0.47	1.91**	84.25**	86.92**	120.55**	2363.93**	761.11**
Hybrids, H	209	0.84**	0.93**	1.50**	1.04**	5.35**	12.60*	14.23**	1339.47**	715.07**
GCA	20	3.99**	4.16**	8.40**	6.33**	26.50**	102.27**	106.97**	8146.64**	4740.95**
SCA	189	0.53**	0.62**	0.83**	0.48**	3.14**	3.12**	4.42**	648.26**	299.01**
HxE	209	0.69**	0.63**	0.86**	0.47**	2.64**	1.74**	2.86**	366.30**	159.28**
GCA × E	20	1.18**	1.97**	2.84**	1.91**	8.48**	2.78**	5.22**	544.96**	160.24**
SCA × E	189	0.61**	0.46*	0.67**	0.32*	2.06**	1.63*	2.62**	324.74**	152.39**
Error	225	0.30	0.32	0.36	0.23	1.23	1.25	1.42	109.10	47.82
Heritability		0.25	0.33	0.41	0.51	0.53	0.84	0.81	0.72	0.82

Note:

** and

* indicate significantly different from zero at 0.01 and 0.1 level of probability, respectively; GCA × E and SCA × E represents general and specific combining ability and their interaction with environments, respectively.

Abbreviations: AD, days to anthesis; DF, degrees of freedom; ED, ear damage; EH, ear height;FD1, FD2, and FD3, mean foliar damage scores at three different time intervals; GCA, specific combining ability; GY, grain yield; PH, plant height; SCA, specific combining ability; SD, days to silkings.

**TABLE 4:** Estimates of genetic parameters for FAW resistance traits, grain yield and other agronomic traits among 210 single cross hybrids evaluated across two environments.

Source	FD1	FD2	FD3	ED	GY	AD	SD	PH	EH
$\sigma^{2}{}_{\text{GCA}}$	0.04**	0.03**	0.07**	0.06**	0.22**	1.29**	1.32**	95.77**	58.34**
$\sigma^{2}{}_{\text{SCA}}$	0.00	0.04**	0.04*	0.04**	0.27**	0.37**	0.45**	80.88**	36.65**
$\sigma^{2}{}_{\text{GCAxE}}$	0.01*	0.04**	0.06**	0.04**	0.17**	0.03**	0.07**	5.80**	0.21*
$\sigma^{2}{}_{\text{SCAxE}}$	0.15	0.07**	0.15*	0.04**	0.39**	0.17**	0.56**	107.72**	52.87**
$\sigma^{2}{}_{\text{e}}$	0.31	0.33	0.37	0.23	1.29	1.30	1.50	109.31	46.66
$\sigma^{2}{}_{\text{A}}$	0.08	0.05	0.14	0.11	0.45	2.58	2.63	191.53	116.69
$\sigma^{2}{}_{\text{D}}$	0.00	0.04	0.04	0.04	0.27	0.37	0.45	80.88	36.65
GCA–SCA ratio	—	0.67	1.79	1.37	0.82	3.48	2.91	1.18	1.59
Baker ratio	1.00	0.60	0.78	0.75	0.62	0.87	0.85	0.70	0.76
$\sigma^{2}{}_{\text{P}}$	0.38	0.42	0.55	0.38	2.01	4.25	4.58	381.72	200.01

Note:

** and

* indicate significantly different from zero at 0.01 and 0.1 probability level, respectively.

Abbreviations: AD, anthesis date; EH, ear height; FD1, FD2 and FD3, mean foliar damage scores at three different time intervals; GY, grain yield; PH, plant height; SD, silking days;$\sigma^{2}{}_{\text{A}}$, $\sigma^{2}{}_{\text{D}}$ and $\sigma^{2}{}_{\text{P}}$ additive, dominance genetic variances and phenotypic variance, respectively;$\sigma^{2}{}_{\text{e}}$, error variance;$\sigma^{2}{}_{\text{GCA}}$, $\sigma^{2}{}_{\text{SCA}}$_, $\sigma^{2}{}_{\text{GCAxE}}$_ and $\sigma^{2}{}_{\text{SCAxE}}$, general and specific combining ability variances and their interaction with environment, respectively.

**TABLE 5:** General combining ability effects of the maize inbred lines for grain yield, FAW resistance traits and other agronomic traits.

Parents	GY	FD1	FD2	FD3	ED	AD	SD	PH	EH	ER
CKSBL10026	−0.63**	−0.08	−0.11*	−0.17**	−0.15**	−0.19*	0.08	−4.03**	5.72**	−2.47**
CKSPL10158	−0.71**	0.31**	0.41**	0.50**	0.04	−0.60**	−1.41**	−3.76**	−10.20**	0.48
CKSPL10007	−0.43**	0.10*	0.27**	0.57**	0.22**	0.12	−0.10	9.45**	−1.89**	4.07**
CKSBL10011	−0.01	−0.10*	−0.09	−0.17**	−0.22**	−1.48**	−1.42**	−0.60	−4.22**	−3.59**
CKSBL10027	−0.87**	−0.17**	−0.20**	−0.37**	0.19**	−0.32**	0.28*	−16.13**	−7.24**	2.45**
CKSBL10039	0.68**	−0.24**	−0.22**	−0.35**	−0.11*	0.52**	0.71**	13.87**	6.26**	−0.32
CKSBL10008	0.81**	−0.15**	−0.20**	−0.07	−0.16**	−0.68**	−0.59**	6.92**	4.59**	−4.02**
CML560	−0.20**	0.27**	0.19**	0.00	−0.02	−0.10	−1.14**	−2.88**	−1.04	−0.63
CKSBL10043	0.12	−0.35**	−0.32**	0.04	−0.22**	−1.73**	−0.71**	−11.61**	−14.42**	−1.92*
CKSBL10060	0.36**	−0.02	−0.02	0.03	−0.25**	−1.77**	−1.64**	−5.35**	−1.10	−2.68**
CKIR04005	0.06	−0.17**	−0.15**	−0.25**	−0.04	−0.22*	−0.35**	−2.62**	0.50	−0.76
CKSBL10153	1.11**	−0.13*	−0.21**	−0.27**	−0.21**	1.49**	1.17**	15.55**	13.15**	−3.94**
CKDHL0214	0.26*	0.34**	0.32**	0.15**	0.33**	1.02**	0.53**	−5.77**	−3.14**	4.85**
CKSBL10020	−1.07**	−0.14**	−0.22**	−0.38**	0.85**	0.15	1.24**	−11.95**	−3.11**	16.88**
CKDHL121320	−0.26*	0.31**	0.07	−0.19**	0.40**	1.64**	1.79**	19.78**	21.76**	6.76**
CLRCY039	0.99**	−0.02	−0.02	−0.19**	−0.30**	2.73**	2.56**	8.71**	4.34**	−3.82**
CKDHL166075	−0.06	−0.07	0.15**	0.45**	0.33**	−0.49**	−0.47**	−10.50**	−5.78**	2.62**
CKDHL164271	0.24*	0.47**	0.39**	0.40**	−0.18**	−0.60**	0.04	1.88*	−0.12	−3.25**
CKSPL10089	0.13	0.03	0.15**	0.43**	−0.17**	−1.24**	−1.94**	13.52**	−3.99**	−2.38**
CML22	−0.01	0.12*	0.18**	0.36**	−0.26**	0.62**	0.09	−1.88**	3.98**	−5.46**
CML71	−0.51**	−0.33**	−0.34**	−0.51**	−0.08	1.15**	1.28**	−12.64**	−4.05**	−2.88**

Note:

* and

** indicate significant at the 0.05 and 0.01 probability level, respectively.

Abbreviations: AD, days to anthesis; ED, ear damage; EH, ear height; ER, number of rotten ears in %; FD1, FD2, FD3, mean foliar damage scores at three time intervals; GY, grain yield; PH, plant height; SD, days to silking.

## Data Availability

All data generated or analysed during this study are included in this manuscript as supplementary material.
