# Supplementary material for: Hybrid breeding for fall armyworm resistance: Combining ability and hybrid prediction
Source: Plant Breed. Author manuscript; Available in PMC 2025 Jan 16. (PMC11737609; doi:10.1111/pbr.13129)
Supplement: Supplementary Figures and Tables [file NIHMS1946405-supplement-Supplementary_Figures_and_Tables.docx]

# Supplementary Materials

# Supplementary Table S1: Number of hybrids evaluated for FAW resistance traits

| No | Hybrid | No | Hybrid | No | Hybrid | No | Hybrid |
| --- | --- | --- | --- | --- | --- | --- | --- |
| 1 | CKIR04005/CKDHL0214 | 54 | CKSBL10060/CKDHL166075 | 107 | CKSPL10158/CKSBL10043 | 160 | CKSBL10060/CLRCY039 |
| 2 | CKDHL0214/CKSBL10008 | 55 | CKSBL10153/CKDHL166075 | 108 | CML560/CKSBL10043 | 161 | CKSBL10153/CLRCY039 |
| 3 | CKSBL10011/CKDHL0214 | 56 | CKSPL10007/CKDHL166075 | 109 | CKSBL10008/CKSBL10060 | 162 | CKSPL10007/CLRCY039 |
| 4 | CKSBL10026/CKDHL0214 | 57 | CKSPL10158/CKDHL166075 | 110 | CKSBL10011/CKSBL10060 | 163 | CKSPL10158/CLRCY039 |
| 5 | CKSBL10027/CKDHL0214 | 58 | CLRCY039/CKDHL166075 | 111 | CKSBL10026/CKSBL10060 | 164 | CML560/CLRCY039 |
| 6 | CKSBL10039/CKDHL0214 | 59 | CML560/CKDHL166075 | 112 | CKSBL10027/CKSBL10060 | 165 | CKDHL0214/CML22 |
| 7 | CKSBL10043/CKDHL0214 | 60 | CKSBL10008/CKIR04005 | 113 | CKSBL10039/CKSBL10060 | 166 | CKDHL121320/CML22 |
| 8 | CKSBL10060/CKDHL0214 | 61 | CKSBL10011/CKIR04005 | 114 | CKSBL10043/CKSBL10060 | 167 | CKDHL164271/CML22 |
| 9 | CKSBL10153/CKDHL0214 | 62 | CKSBL10026/CKIR04005 | 115 | CKSPL10007/CKSBL10060 | 168 | CKDHL166075/CML22 |
| 10 | CKSPL10007/CKDHL0214 | 63 | CKSBL10027/CKIR04005 | 116 | CKSPL10158/CKSBL10060 | 169 | CKIR04005/CML22 |
| 11 | CKSPL10158/CKDHL0214 | 64 | CKSBL10039/CKIR04005 | 117 | CML560/CKSBL10060 | 170 | CKSBL10008/CML22 |
| 12 | CML560/CKDHL0214 | 65 | CKSBL10043/CKIR04005 | 118 | CKIR04005/CKSBL10153 | 171 | CKSBL10011/CML22 |
| 13 | CKDHL0214/CKDHL121320 | 66 | CKSBL10060/CKIR04005 | 119 | CKSBL10008/CKSBL10153 | 172 | CKSBL10020/CML22 |
| 14 | CKIR04005/CKDHL121320 | 67 | CKSPL10007/CKIR04005 | 120 | CKSBL10011/CKSBL10153 | 173 | CKSBL10026/CML22 |
| 15 | CKSBL10008/CKDHL121320 | 68 | CKSPL10158/CKIR04005 | 121 | CKSBL10026/CKSBL10153 | 174 | CKSBL10027/CML22 |
| 16 | CKSBL10011/CKDHL121320 | 69 | CML560/CKIR04005 | 122 | CKSBL10027/CKSBL10153 | 175 | CKSBL10039/CML22 |
| 17 | CKSBL10020/CKDHL121320 | 70 | CKSBL10011/CKSBL10008 | 123 | CKSBL10039/CKSBL10153 | 176 | CKSBL10043/CML22 |
| 18 | CKSBL10026/CKDHL121320 | 71 | CKSBL10008/CKSBL10026 | 124 | CKSBL10043/CKSBL10153 | 177 | CKSBL10060/CML22 |
| 19 | CKDHL121320/CKSBL10027 | 72 | CKSBL10027/CKSBL10008 | 125 | CKSBL10060/CKSBL10153 | 178 | CKSBL10153/CML22 |
| 20 | CKSBL10039/CKDHL121320 | 73 | CKSBL10039/CKSBL10008 | 126 | CKSPL10007/CKSBL10153 | 179 | CKSPL10007/CML22 |
| 21 | CKSBL10043/CKDHL121320 | 74 | CKSPL10007/CKSBL10008 | 127 | CKSPL10158/CKSBL10153 | 180 | CKSPL10089/CML22 |
| 22 | CKSBL10060/CKDHL121320 | 75 | CKSPL10158/CKSBL10008 | 128 | CML560/CKSBL10153 | 181 | CKSPL10158/CML22 |
| 23 | CKSBL10153/CKDHL121320 | 76 | CKSBL10026/CKSBL10011 | 129 | CKSBL10026/CKSPL10007 | 182 | CLRCY039/CML22 |
| 24 | CKSPL10007/CKDHL121320 | 77 | CKSPL10007/CKSBL10011 | 130 | CKSPL10158/CKSPL10007 | 183 | CML560/CML22 |
| 25 | CKSPL10158/CKDHL121320 | 78 | CKSPL10158/CKSBL10011 | 131 | CKDHL0214/CKSPL10089 | 184 | CML560/CKSBL10008 |
| 26 | CML560/CKDHL121320 | 79 | CKSBL10020/CKDHL0214 | 132 | CKDHL121320/CKSPL10089 | 185 | CKSBL10011/CML560 |
| 27 | CKDHL0214/CKDHL164271 | 80 | CKIR04005/CKSBL10020 | 133 | CKDHL164271/CKSPL10089 | 186 | CKSBL10026/CML560 |
| 28 | CKDHL121320/CKDHL164271 | 81 | CKSBL10008/CKSBL10020 | 134 | CKDHL166075/CKSPL10089 | 187 | CKSBL10027/CML560 |
| 29 | CKDHL166075/CKDHL164271 | 82 | CKSBL10011/CKSBL10020 | 135 | CKIR04005/CKSPL10089 | 188 | CKSBL10039/CML560 |
| 30 | CKIR04005/CKDHL164271 | 83 | CKSBL10026/CKSBL10020 | 136 | CKSBL10008/CKSPL10089 | 189 | CKSPL10007/CML560 |
| 31 | CKSBL10008/CKDHL164271 | 84 | CKSBL10027/CKSBL10020 | 137 | CKSBL10011/CKSPL10089 | 190 | CKSPL10158/CML560 |
| 32 | CKSBL10011/CKDHL164271 | 85 | CKSBL10039/CKSBL10020 | 138 | CKSBL10020/CKSPL10089 | 191 | CKDHL0214/CML71 |
| 33 | CKSBL10020/CKDHL164271 | 86 | CKSBL10043/CKSBL10020 | 139 | CKSBL10026/CKSPL10089 | 192 | CKDHL121320/CML71 |
| 34 | CKSBL10026/CKDHL164271 | 87 | CKSBL10060/CKSBL10020 | 140 | CKSBL10027/CKSPL10089 | 193 | CKDHL164271/CML71 |
| 35 | CKSBL10027/CKDHL164271 | 88 | CKSBL10153/CKSBL10020 | 141 | CKSBL10039/CKSPL10089 | 194 | CKDHL166075/CML71 |
| 36 | CKSBL10039/CKDHL164271 | 89 | CKSPL10007/CKSBL10020 | 142 | CKSBL10043/CKSPL10089 | 195 | CKIR04005/CML71 |
| 37 | CKSBL10043/CKDHL164271 | 90 | CKSPL10158/CKSBL10020 | 143 | CKSBL10060/CKSPL10089 | 196 | CKSBL10008/CML71 |
| 38 | CKSBL10060/CKDHL164271 | 91 | CML560/CKSBL10020 | 144 | CKSBL10153/CKSPL10089 | 197 | CKSBL10011/CML71 |
| 39 | CKSBL10153/CKDHL164271 | 92 | CKSBL10011/CKSBL10027 | 145 | CKSPL10007/CKSPL10089 | 198 | CKSBL10020/CML71 |
| 40 | CKSPL10007/CKDHL164271 | 93 | CKSBL10026/CKSBL10027 | 146 | CKSPL10158/CKSPL10089 | 199 | CKSBL10026/CML71 |
| 41 | CKSPL10158/CKDHL164271 | 94 | CKSPL10007/CKSBL10027 | 147 | CLRCY039/CKSPL10089 | 200 | CKSBL10027/CML71 |
| 42 | CLRCY039/CKDHL164271 | 95 | CKSPL10158/CKSBL10027 | 148 | CML560/CKSPL10089 | 201 | CKSBL10039/CML71 |
| 43 | CML560/CKDHL164271 | 96 | CKSBL10011/CKSBL10039 | 149 | CKSBL10026/CKSPL10158 | 202 | CKSBL10043/CML71 |
| 44 | CKDHL0214/CKDHL166075 | 97 | CKSBL10026/CKSBL10039 | 150 | CKDHL0214/CLRCY039 | 203 | CKSBL10060/CML71 |
| 45 | CKDHL121320/CKDHL166075 | 98 | CKSBL10027/CKSBL10039 | 151 | CKDHL121320/CLRCY039 | 204 | CKSBL10153/CML71 |
| 46 | CKIR04005/CKDHL166075 | 99 | CKSPL10007/CKSBL10039 | 152 | CKIR04005/CLRCY039 | 205 | CKSPL10007/CML71 |
| 47 | CKSBL10008/CKDHL166075 | 100 | CKSPL10158/CKSBL10039 | 153 | CKSBL10008/CLRCY039 | 206 | CKSPL10089/CML71 |
| 48 | CKSBL10011/CKDHL166075 | 101 | CKSBL10008/CKSBL10043 | 154 | CKSBL10011/CLRCY039 | 207 | CKSPL10158/CML71 |
| 49 | CKSBL10020/CKDHL166075 | 102 | CKSBL10011/CKSBL10043 | 155 | CKSBL10020/CLRCY039 | 208 | CLRCY039/CML71 |
| 50 | CKSBL10026/CKDHL166075 | 103 | CKSBL10026/CKSBL10043 | 156 | CKSBL10026/CLRCY039 | 209 | CML22/CML71 |
| 51 | CKSBL10027/CKDHL166075 | 104 | CKSBL10027/CKSBL10043 | 157 | CKSBL10027/CLRCY039 | 210 | CML560/CML71 |
| 52 | CKSBL10039/CKDHL166075 | 105 | CKSBL10039/CKSBL10043 | 158 | CKSBL10039/CLRCY039 |  |  |
| 53 | CKSBL10043/CKDHL166075 | 106 | CKSPL10007/CKSBL10043 | 159 | CKSBL10043/CLRCY039 |  |  |

Supplementary Table S2**.** Phenotypic correlations among test locations for resistances against FAW based on evaluation of 241 F1 hybrids.

| *Locations Trait | Correlations (r_P_) |
| --- | --- |
| Kbk1GY_vs_Kkm2GY | 0.39** |
| Kbk1ER_vs_Kkm2ER | 0.28** |
| Kbk1AD_vs_Kkm2AD | 0.81** |
| Kbk1SD_vs_Kkm2SD | 0.76** |
| Kbk1ASI_vs_Kkm2ASI | 0.57** |
| Kbk1PH_vs_Kkm2PH | 0.70** |
| Kbk1EH_vs_Kkm2EH | 0.77** |
| Kbk1FD1_vs_Kkm2FD1 | 0.18** |
| Kbk1FD2_vs_Kkm2FD2 | 0.29** |
| Kbk1FD3_vs_Kkm2FD3 | 0.29** |
| Kbk1ED_vs_Kkm2ED | 0.40** |
| Kbk1EPO_vs_Kkm2EPO | 0.74** |
| Kbk1Moi_vs_Kkm2Moi | 0.18** |

*Kbk1 – Kiboko, Kkm2 – Kakamega, AD, anthesis date, ASI, anthesis to silking interval; ED, ear damage; EH, ear height; EPO, ear position; ER, ear rot; FD1, FD2, FD3, mean leaf damage scores 7,14 and 21 days after FAW infestation, respectively; Moi, Moisture; PH, plant height; SD, silking date. ** significantly different from zero at 0.01 level of probability


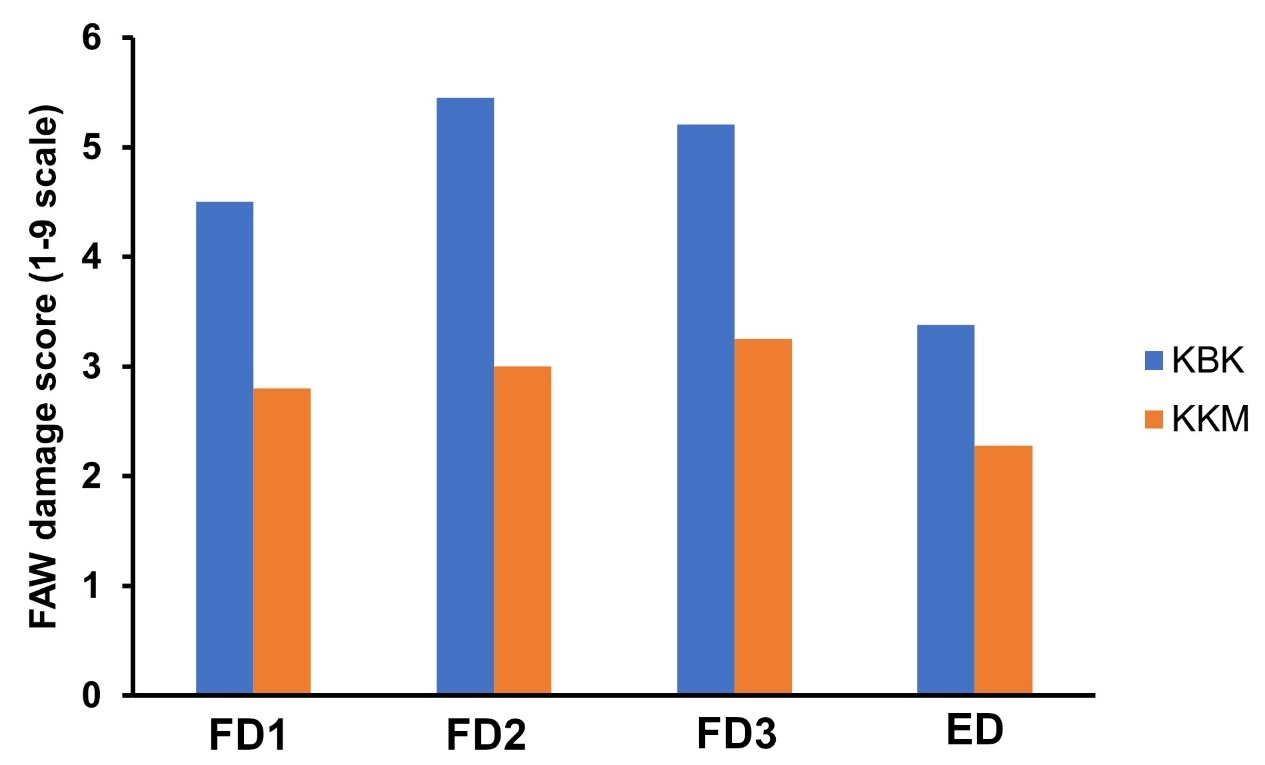


**Supplementary Figure S1**. Mean performance of 241 F1 hybrids for foliar damage (FD) and ear damage (ED) on a scale from 1 (fully resistant) to 9 (fully susceptible) in Kiboko (KBK) and Kakamega (KKM) locations.
